# A Rare Case of Leiomyosarcoma Arising From the Stomach: A Case Report and Review of the Literature

**DOI:** 10.7759/cureus.59406

**Published:** 2024-04-30

**Authors:** Andrew J Gonedes, Keshav Bhattar, Jeffrey Valencia Uribe, Andres Reyes-Corcho, Eric Boccio

**Affiliations:** 1 Emergency Medicine, Memorial Healthcare System, Pembroke Pines, USA; 2 Internal Medicine, Memorial Healthcare System, Pembroke Pines, USA

**Keywords:** endoloop ligation, genomic instability, surgical-wide excision, gastrointestinal stromal tumor, leiomyosarcoma

## Abstract

Leiomyosarcomas (LMSs) account for 10-20% of all soft-tissue sarcomas (STSs). Soft-tissue sarcomas, and more specifically LMS, typically originate from the uterus, extremity, retroperitoneal, or lower intraabdominal gastrointestinal organs. Due to the rarity and variability in presentation, it is difficult to describe identifiable risk factors, determine etiology, predict disease progression, and prognosticate these types of neoplasms. We present the case of a 77-year-old woman presenting to the emergency department with shortness of breath. After being diagnosed and treated for mild exacerbation of congestive heart failure, she was incidentally found to be anemic. Further workup, including an esophagogastroduodenoscopy, revealed a bleeding gastric mass, which was biopsied. Histopathology and immunohistochemistry confirmed the mass to be primary gastric LMS. Due to its rarity, an interdisciplinary approach involving clinical, histopathologic, and immunohistochemical data is necessary to successfully identify and diagnose gastrointestinal LMS. This case report aims to contribute to the paucity of information available in the literature regarding gastric LMS so that it may be better understood.

## Introduction

Soft-tissue sarcomas (STSs) originate mainly from the embryonic mesoderm and are a rare form of malignancy, accounting for less than one percent of all adult cancers. Leiomyosarcomas (LMSs) comprise up to 10-20% of all STSs and exhibit varying responses and sensitivities to therapy based on the organ of origin [1-2]. While the majority of cases involve the abdominopelvic organs, LMSs also arise within the retroperitoneum, large blood vessels, and extremities with a preference for the thigh [2]. The majority of gastrointestinal LMSs involve the lower tract, originating in the small intestine or colorectum. Approximately 10% of gastrointestinal LMSs are esophageal or gastric in origin [2-3]. Risk factors for LMS include a prior history of radiotherapy, genetic syndromes including hereditary retinoblastoma and Li-Fraumeni syndrome, and Tamoxifen use [1,4-5]. Patients suffering from gastrointestinal LMS typically present with abdominal pain or discomfort, bloating, unintentional weight loss, changes in bowel habits, symptomatic and incidental anemia, and gross or occult gastrointestinal bleeding [3,6].

## Case presentation

**Figure 1:**
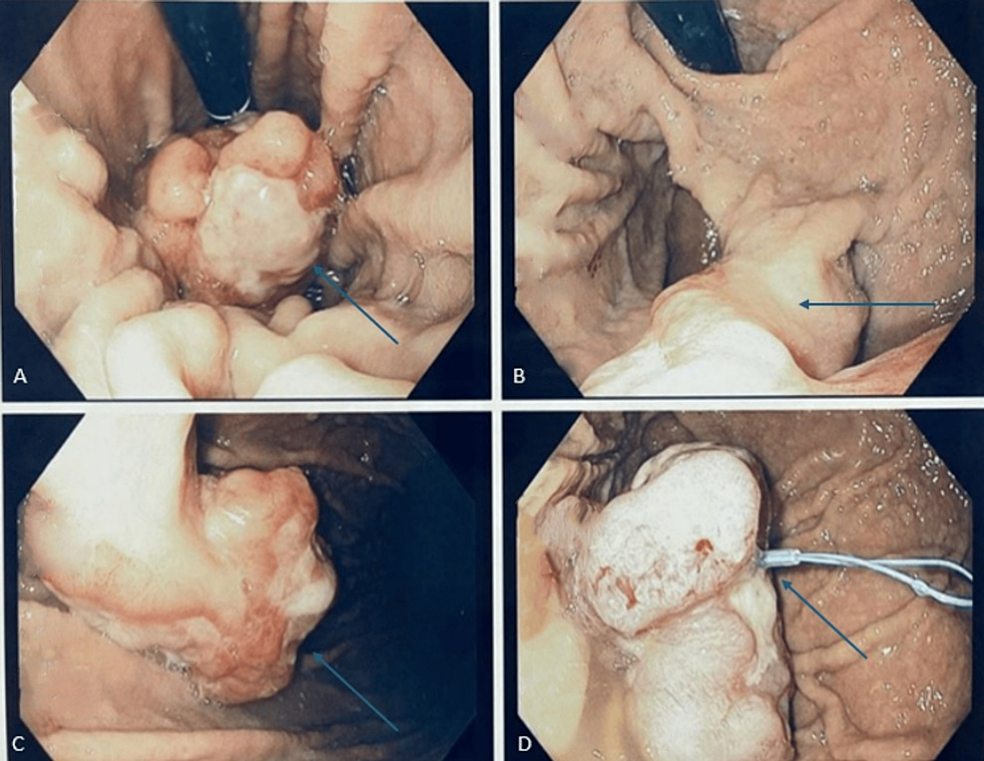
Images collected during esophagogastroduodenoscopy (EGD) A-C) Normal esophagus with a large 4.1 centimeters pedunculated, ulcerating lesion at the proximal greater curvature of the stomach (blue arrows). D) Endoloop deployed around the middle of the lesion to facilitate resection and specimen recovery (blue arrow).

A 77-year-old female with a past medical history of type 2 diabetes, hyperlipidemia, atrial fibrillation on apixaban, heart failure with preserved ejection fraction, severe anemia, and interstitial lung disease presented to the emergency department (ED) with progressive dyspnea. Diagnostic workup in the ED revealed the patient was suffering from a mild exacerbation of congestive heart failure, and her symptoms noticeably improved following the administration of intravenous loop diuretics. The patient was also noted to be anemic with hemoglobin at 8.2 g/dL consistent with previous laboratory results and the list of active medical problems. The family reported that the patient had recently been hospitalized at an outside facility for severe anemia; at that time, esophagogastroduodenoscopy (EGD) was performed, which revealed a gastric mass, and pathology was consistent with high-grade dysplasia. During this hospitalization, gastroenterology was consulted, and repeat EGD revealed a large 5.1 cm pedunculated, ulcerating gastric mass at the proximal greater curvature. An endoloop was deployed, and a portion of the lesion was endoscopically resected (Figure 1).

**Figure 2:**
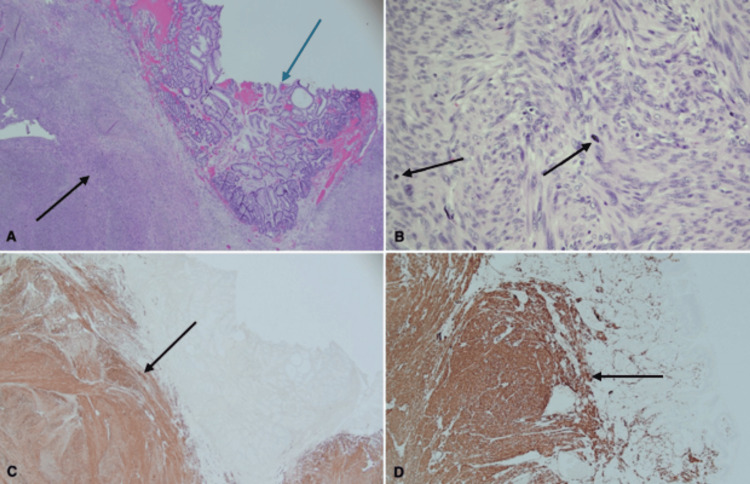
Histopathology and immunohistochemistry slides from recovered gastric mass specimen A) Low power view of the overlying gastric mucosa (blue arrow) with submucosa replaced by leiomyosarcoma (black arrow). B) High power view demonstrating pleomorphic nuclei and increased mitotic activity (black arrows). C) Desmin positive staining of submucosal cells (black arrow). D) Smooth muscle actin positive staining of submucosal cells (black arrow).

Biopsy of the lesion was consistent with ulcerated gastric mucosa and separate detached fragments of granulation tissue exhibiting severe acute inflammation. The pathology report indicated that the specimen was organ-confined with no involvement of the serosal surface. Histopathology revealed a spindle cell neoplasm, later identified to be leiomyosarcoma, AJCC pathologic stage: pT1. The atypical spindle cells demonstrated cytologic atypia and increased mitotic activity with foci of marked nuclear pleomorphism. Ki-67 staining revealed an increased proliferative index. The spindle cells were negative for CD117, DOG1, CD34, and S100, but diffusely positive for desmin and smooth muscle actin, supporting the diagnosis of leiomyosarcoma (Figure 2). Tumor markers like carcinoembryonic antigen (CEA), alpha-fetoprotein (AFP), and Cancer Antigen-125 (CA-125) were all within normal limits. Computed tomography (CT) of the chest without intravenous contrast revealed no evidence of metastases.

The patient subsequently underwent robot-assisted partial gastrectomy. The pathology report of the resected gastric mass was consistent with LMS; all margins of resection were negative, and the deep margin consisted of benign gastric mucosa. No adjuvant therapy was recommended. The patient recovered and was discharged on postoperative day 3 with a scheduled surveillance CT chest and a follow-up visit in 6 months.

## Discussion

Descriptions of gastric LMSs in the literature remain sparse, and very few epidemiological studies have been reported. A retrospective observational study that included 523 patients with gastrointestinal LMS calculated the average age of presentation to be 66 years. Seventy-eight percent of patients were white, and the incidence amongst men and women was similar. Sites of origin included the small intestine (31.7%), stomach (28.3%), colon (26.4%), rectum (9.4%), and esophagus (4.2%), and there were no significant differences between tumor sites for age, gender, and race [7]. According to a case review involving 14 cases of gastric LMS, age and portion of the stomach involved were not associated; nine cases (64%) were alive at the end of the 10 to 36-month follow-up period, four (29%) had metastatic disease on presentation, eleven (79%) were treated by total or subtotal gastrectomy, and only two (14%) received chemotherapy [8].

Patients with gastric LMS commonly present with epigastric abdominal pain, described as dull or crampy. Gastrointestinal bleeding may manifest as melena or hematemesis, reflecting the tumor's propensity to ulcerate and invade blood vessels [9]. Anorexia, unintentional weight loss, and fatigue are frequent constitutional symptoms associated with advanced stages of the disease. Metastatic gastric LMS is rare as the tumor progresses slowly and produces noticeable symptoms [2-3]. The lack of distinctive clinical features underscores the importance of heightened awareness and clinical suspicion, diagnostic imaging, endoscopy and specimen recovery, and histology. A formal lymph node dissection is not required as lymphatic spread is uncommon. Histopathological testing is crucial to ensure timely diagnosis and referral to specialty care.

A CT scan can better visualize retroperitoneal and visceral lesions for LMS of abdominopelvic origin. For LMS arising within the extremities, head, or neck, magnetic resonance imaging (MRI) is the preferred imaging modality [10]. Due to its hematogenous mode of metastasis, spread to the lung and liver needs to be ruled out. For uterine masses, a core needle biopsy or an open-incisional biopsy is necessary for confirmation of the diagnosis. Endoscopic ultrasonography can be utilized for upper gastrointestinal lesions smaller than 2 cm. For larger gastric LMS, gastrectomy is often required and is both diagnostic and therapeutic.

Histologically, LMS is classified into spindle and non-spindle morphology; the latter has been associated with a poorer prognosis for uterine LMS [11-12]. Prior to the development of c-Kit immunohistochemistry, gastrointestinal stromal tumors (GISTs) of the stomach were often described as LMSs. GISTs originate from the interstitial cells of Cajal but exhibit gross and microscopic morphology similar to LMSs. However, the incidence of primary LMSs of gastric origin is very low. LMSs are distinguished from GISTs based on their positivity for smooth muscle actin (SMA) or desmin, and their negativity for CD117, DOG1, and CD34. It is crucial to distinguish LMSs from GISTs since only the latter responds to c-Kit-targeted therapies such as imatinib or sunitinib, and expresses intrinsic resistance to many conventional anticancer drugs due to their production of drug-resistance proteins MDR1 and MRP1 [9, 13-14]. LMS usually has inconsistent, complex, and unbalanced karyotypes with extreme genomic instability, making it a very heterogeneous disease. Most notable associations include loss in chromosomes 10q (PTEN) and 13q (RB1), and gain at 17p (TP53) [1].

Per the 2022 National Comprehensive Cancer Network guidelines for STS, treatment for nonmetastatic primary retroperitoneal and intraabdominal sarcomas that are potentially curative involves a surgical wide local excision with a microscopic negative margin [15]. A formal lymph node dissection is not required as lymphatic spread is uncommon. Multimodal therapy involving radiotherapy (RT) and chemotherapy is being investigated and is currently recommended for cases with high local recurrence rates and inability to achieve negative surgical margins [15]. For lesions amenable to resection, decisions regarding adjuvant or neoadjuvant chemotherapy or RT are made on a case-by-case basis per clinical judgment. For unresectable diseases, defined as the involvement of vital organs or structures whose removal would cause unacceptable morbidity, a biopsy is recommended prior to treatment with systemic chemotherapy or RT. In patients with metastatic disease, surgical resection is often recommended for palliative purposes [15]. Given that the recovered specimen demonstrated benign margins with deep margins consistent with normal gastric mucosa, the patient referenced in the case report underwent wide local excision, and no adjuvant therapy was recommended.

For cases in which chemotherapy is indicated, anthracycline-based regimens are most favored [16]. A combination of doxorubicin and ifosfamide has been used for treating LMSs. Uterine and metastatic LMS are sensitive to gemcitabine monotherapy, and gemcitabine and docetaxel combination therapy [17]. Second-line therapies include trabectedin, pazopanib, and eribulin. Trabectedin is a marine alkaloid that interferes with transcription and DNA repair. Pazopanib inhibits multiple tyrosine kinases, including VEGFR-1, VEGFR- 2, VEGFR-3, PDGFR-α, PDGFR-β, FGFR-1, FGFR-3, c-Kit, IL-2 receptor-inducible T-cell kinase, leukocyte-specific protein tyrosine kinase, and transmembrane glycoprotein receptor tyrosine kinase (c-fms) [18]. Immunotherapy with agents such as nivolumab and pembrolizumab has also been used, sometimes in combination with ipilimumab, but this combination has not received FDA approval [19]. Currently, several phase I and II drugs are being investigated and developed in conjunction with radiation and chemotherapy [1]. The Eastern Cooperative Oncology Group and the Southwest Oncology Group report response rates to chemotherapy at 20-25% for uterine leiomyosarcomas, which is double the response rates for gastrointestinal LMSs. However, the response rate of gastrointestinal LMSs is most likely unknown, as many of these cases were likely misclassified as GISTs given that c-Kit immunohistochemistry testing was unavailable [13].

Surveillance imaging involving CT or MRI of the chest, abdomen, and pelvis is recommended every 3 to 6 months for 2 to 3 years, then every 6 months for the next 2 years, and then annually [15]. The patient in the case presented is scheduled for surveillance CT imaging and a follow-up visit in 6 months. The prognosis varies among patients who undergo surgery and those who do not. The 5-year cancer-specific survival and overall survival rates have been reported at 92.5% and 84.8%, respectively, for patients who undergo surgical management compared to 61.3% and 79%, respectively, for those who do not undergo surgical resection [2-3]. Prognostication for gastric LMS is difficult given its rarity and assumed underdiagnosis and misclassification. Prognostic data for gastric LMS is extrapolated from LMS originating outside of the gastrointestinal tract or GISTs that may have been misidentified as LMS.

## Conclusions

Gastrointestinal LMS originating from the stomach remains poorly understood, with few reported cases and variable clinical presentations and courses. Our case report underscores the clinical challenge posed by gastric LMS. The symptomatic overlap and pathologic similarities with GISTs necessitate a comprehensive evaluation, and immunohistochemistry focused on c-Kit expression has emerged as a pivotal diagnostic tool to precisely differentiate GISTs from LMSs. Recognition of this diagnostic dilemma underscores the significance of adopting a broad differential framework in assessing gastric mesenchymal tumors, which ultimately influences therapeutic management and prognosis.
